# Beneficial Effects of Adjusted Perfusion and Defibrillation Strategies on Rhythm Control within Controlled Automated Reperfusion of the Whole Body (CARL) for Refractory Out-of-Hospital Cardiac Arrest

**DOI:** 10.3390/jcm11082111

**Published:** 2022-04-11

**Authors:** Sam Joé Brixius, Jan-Steffen Pooth, Jörg Haberstroh, Domagoj Damjanovic, Christian Scherer, Philipp Greiner, Christoph Benk, Friedhelm Beyersdorf, Georg Trummer

**Affiliations:** 1 Department of Cardiovascular Surgery, Faculty of Medicine, University Medical Centre Freiburg, University of Freiburg, 79106 Freiburg, Germany; jan-steffen.pooth@uniklinik-freiburg.de (J.-S.P.); domagoj.damjanovic@uniklinik-freiburg.de (D.D.); christian.scherer@uniklinik-freiburg.de (C.S.); greiner-philipp@uniklinik-freiburg.de (P.G.); christoph.benk@uniklinik-freiburg.de (C.B.); friedhelm.beyersdorf@uniklinik-freiburg.de (F.B.); georg.trummer@uniklinik-freiburg.de (G.T.); 2 Centre for Experimental Models and Transgenic Service, Department of Experimental Surgery, Faculty of Medicine, University Medical Centre Freiburg, University of Freiburg, 79104 Freiburg, Germany; joerg.haberstroh@uniklinik-freiburg.de

**Keywords:** cardiopulmonary resuscitation, extracorporeal circulation, post-resuscitation care

## Abstract

Survival and neurological outcomes after out-of-hospital cardiac arrest (OHCA) remain low. The further development of prehospital extracorporeal resuscitation (ECPR) towards Controlled Automated Reperfusion of the Whole Body (CARL) has the potential to improve survival and outcome in these patients. In CARL therapy, pulsatile, high blood-flow reperfusion is performed combined with several modified reperfusion parameters and adjusted defibrillation strategies. We aimed to investigate whether pulsatile, high-flow reperfusion is feasible in refractory OHCA and whether the CARL approach improves heart-rhythm control during ECPR. In a reality-based porcine model of refractory OHCA, 20 pigs underwent prehospital CARL or conventional ECPR. Significantly higher pulsatile blood-flow proved to be feasible, and critical hypotension was consistently prevented via CARL. In the CARL group, spontaneous rhythm conversions were observed using a modified priming solution. Applying potassium-induced secondary cardioplegia proved to be a safe and effective method for sustained rhythm conversion. Moreover, significantly fewer defibrillation attempts were needed, and cardiac arrhythmias were reduced during reperfusion via CARL. Prehospital CARL therapy thus not only proved to be feasible after prolonged OHCA, but it turned out to be superior to conventional ECPR regarding rhythm control.

## 1. Introduction

Sudden out-of-hospital cardiac arrest (OHCA) is one of the leading causes of death in Europe and a major health care problem [1,2]. Its survival rate is still reported to be poor, with a significant number of patients suffering from permanent neurological disability [1,3,4,5]. In recent years, extracorporeal cardiopulmonary resuscitation (ECPR) has shown promising results and consequentially found consideration in the guidelines as a rescue therapy in refractory cardiac arrest (CA) for selected patients [6,7,8].

However, uncontrolled reperfusion leads to the development of so-called ischaemia-reperfusion injury (IRI), causing further multi-organ damage [9,10]. The pathophysiologic mechanisms of IRI include systemic inflammatory response, oxidative stress and microcirculatory deficits [9,11]. In particular, cardiac IRI often interferes with the aim of re-establishing a regular heart rhythm. As a result, cardiac arrhythmias are frequently detected during ECPR. Early rhythm conversion and termination of these arrhythmias reduce myocardial oxygen consumption and are essential to unloading the left ventricle [12,13]. As a result, the exacerbation of myocardial ischaemic injury and potential side effects of cardiac standstill during extracorporeal circulation (ECC), such as left ventricle distension or intra-cardiac thrombus formation, can be prevented [14,15]. Early restoration of a regular cardiac rhythm and preventing further cardiac arrhythmias should therefore be among the highest priorities during ECPR.

To further improve the outcome after CA, a concept based on ECC has been developed, called Controlled Automated Reperfusion of the Whole Body (CARL). CARL enables users to treat and minimise IRI by controlling the initial reperfusion phase and applying basic scientific principles. Its rationale has been reported [9,16,17,18]. In addition to acquiring and modifying several reperfusion parameters, reperfusion with high, pulsatile blood flow and high reperfusion pressures are key elements of CARL therapy. Based on this, an adapted approach to rhythm conversion using cardiac surgery techniques such as secondary cardioplegia (SC) is followed here [16,19,20].

Our first study aim was to investigate whether a pulsatile, high blood flow approach is feasible despite expected hypovolemia and vasoplegia during refractory cardiopulmonary resuscitation. Second, we wanted to investigate whether modified reperfusion targets when applying CARL therapy have any effects on the rhythm conversion rate and heart rhythm control in comparison to conventional ECPR.

## 2. Materials and Methods

This animal study protocol was approved by the local animal welfare committee (Regierungspräsidium Freiburg, G-15/148). All experiments were carried out in accordance with the regulations of the German animal protection law and animal care guidelines of the European Community (2010/63/EU).

**Figure 1:**
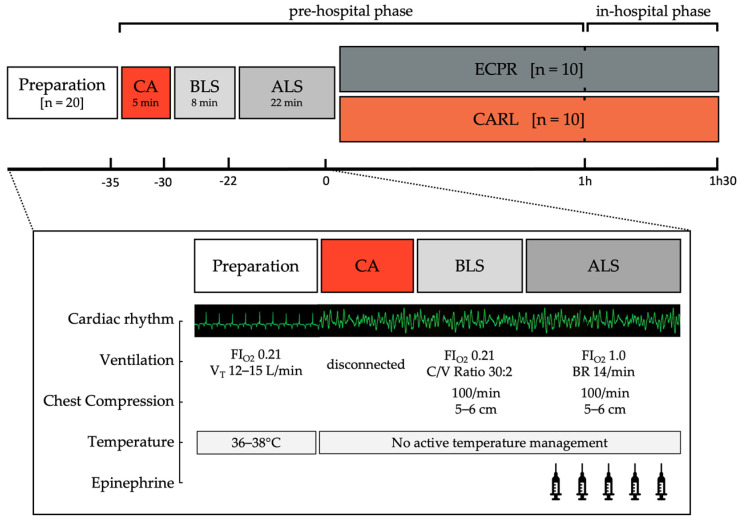
Schematic presentation of the experimental protocol. CA Cardiac arrest, BLS Basic life support, ALS Advanced life support, FiO_2_ Fraction of inspired oxygen, V_T_ Tidal volume, C/V Chest compression to ventilation ratio, BR Breathing rate.

To create a reality-based porcine model of refractory OHCA, local response times for OHCA were included in our study protocol. For this purpose, the experimental procedure proceeded realistically in individual stages (see Figure 1), starting with a normothermic whole-body ischaemic phase. In this stage, bystanders witness the collapse, CA is observed, and the emergency medical services are dispatched (5 min). In the second stage, basic life support is attempted via high quality chest compression and rescue breaths (8 min). When the specialized rescue forces arrive, additional rescue attempts and advanced life support are started (22 min).

A total of 35 min after the CA onset, the therapy is escalated to one of the extracorporeal systems (conventional ECPR versus CARL). The extracorporeal resuscitation phase was further divided into two sections: a pre-hospital and an in-hospital section.

The preparations and realization of each phase according to the group’s allocation are explained in detail below.

### 2.1. Anaesthesia and Surgical Procedures

In our experimental protocol, 20 Landrace-Hybrid pigs of 50–75 kg bodyweight were premedicated with ketamine (20 mg/kg) and midazolam (5 mg/kg) intramuscularly. Anaesthesia was induced by injecting propofol (2–4 mg/kg) through a venous catheter placed in the marginal ear vein. After neuromuscular relaxation with vecuronium (10 mg), endotracheal intubation was performed with an endotracheal tube (size 7.0–8.0 mm). Volume-controlled mechanical ventilation was provided to normalize pH, arterial partial pressure of oxygen (P_O2_) and arterial partial pressure of carbon dioxide (P_CO2_). Anaesthesia was maintained with inhaled isoflurane (2% Volume), fentanyl (1–5 μg/kg/h) and vecuronium (0.2 mg/kg/h). Hypokalaemia and hypomagnesemia, as routinely observed in prior experiments, were compensated accordingly with 5 mL potassium chloride 7.45% and 1 g magnesium. An antibiotic prophylaxis was provided with ceftriaxone (2 g). Ringer’s solution (10 mL/kg/h) was used as primary fluid substitution to maintain euvolemia. We employed a continuous intraoperative monitoring, which recorded heart rate, pulse strength, oxygen saturation, ECG and blood pressure automatically (Siemens, SC 9000XL, Washington, DC, USA). Body temperature was recorded via a nasopharyngeal temperature probe and kept constant in the physiological range until the experimental protocol was started.

Animals were placed in a horizontal supine position on a specially designed resuscitation board with a built-in connection point for the mechanical resuscitation device [21]. The neck vessels were exposed by chirurgical dissection on the right side of the neck. Two catheters were placed in the right common carotid artery to continuously record the arterial blood pressure and collect arterial blood samples. Another catheter was placed in the external jugular vein for venous blood sampling. Information on pulmonary arterial and central venous pressures as well as cardiac output was obtained via a Swan–Ganz catheter (Swan-Ganz Pacing-TD Catheter, Edwards Lifesciences, Irvine, CA, USA), which was placed in the right subclavian vein. Coronary perfusion pressure was calculated as the difference between the diastolic and central venous pressure [22]. The right femoral artery and right external jugular vein were prepared for later cannulation.

At fixed time points during the experiment, blood samples were taken for arterial and venous blood gas analyses.

### 2.2. Cardiac Arrest

Cardiac arrest was induced by electrical fibrillation using the pacing electrodes of the Swan–Ganz catheter. The so induced cardiac arrest was kept for the next five minutes. At the same time the animals were disconnected from mechanical ventilation. During this phase, the supply of all drugs was stopped, and no resuscitation of any kind took place.

To prepare for extracorporeal circulation, a 22–24 F cannula was advanced in the exposed right jugular vein for venous drainage, as was a 14–16 F cannula in the right femoral artery for arterial inflow. Each cannula was flushed with saline and 5000 I.U. of heparin to prevent clot formation; they remained clamped during the CA and CPR periods.

### 2.3. Basic Life Support

Basic life support (BLS) was provided for eight minutes in accordance with the BLS algorithm of the European Resuscitation Council (ERC) [23]. Chest compressions were performed using a mechanical chest compression device (LUCAS 2 Chest Compression Device, Stryker Medical, Portage, MI, USA) at a frequency of 100 compressions per minute and compression depth of 5–6 cm. The time ratio between compression and decompression was 50/50, and active decompression was applied. The animals were manually ventilated with ambient air (21% oxygen) by connecting the endotracheal tube to a ventilation bag. The chest compression to ventilation ratio was 30 to 2.

### 2.4. Advanced Life Support

In the subsequent advanced life support (ALS) phase, the inspiratory oxygen concentration was raised to 100% and chest compressions were no longer interrupted for ventilation. Ventilation occurred at a rate of 14/min with a tidal volume of 10 mL/kg. In accordance with ERC guidelines, each animal received 1 mg epinephrine (10 mL diluted 1:10 with NaCl 0.9%) every 4 min via intravenous access. Cumulatively, each animal received 5 mg of epinephrine flushed with 20 mL of saline solution. Electrical or chemical defibrillation attempts were not made throughout the duration of conventional CPR to simulate refractory CA and prevent a premature return of spontaneous circulation (ROSC). Anaesthesia was resumed with propofol (10–15 mg/kg/h), fentanyl (1–5 μg/kg/h) and vecuronium (0.2 mg/kg/h) to prevent awareness.

### 2.5. Animal Groups

Following five minutes of normothermic CA and 30 min of conventional resuscitation, ECC was established for 90 min according to a group-specific protocol. n = 10 pigs were allocated to each group. In both groups, chest compressions were stopped after establishing ECC, and rhythm conversion to a regular heart rhythm was attempted. Any cardiac arrhythmias during reperfusion were treated when hemodynamically relevant.

#### 2.5.1. Conventional Extracorporeal Cardiopulmonary Resuscitation (ECPR)

The selected reperfusion targets and settings in the group receiving conventional ECPR were realized just as they are already practiced in OHCA in many places. ECC was established using a diagonal pump (Deltastream DP3, Medos Medizintechnik AG, Stolberg, Germany) and a commercially available reperfusion set (Medos Medizintechnik AG, Stolberg, Germany). The system was primed with a total of 1.2 L crystalloid solution (Jonosteril Infusion Solution, Fresenius Kabi Deutschland GmbH, Bad Homburg, Germany) at room temperature and 15,000 I.U. heparin. Reperfusion was performed with a blood flow of 50 mL/kg/min and sweep gas flow of 2.5–3.5 L/min with 100% oxygen supply via the membrane oxygenator. The inspiratory oxygen concentration was set to 100% and ventilation was continued with a tidal volume of 10 mL/kg/min.

Persistent shockable cardiac arrhythmias were treated in accordance with the ALS-algorithm [6]. If defibrillation was indicated, up to three shocks were applied at an energy of 200 J followed by an initial dose of amiodarone (10 mg/kg) if unsuccessful. A second dose of amiodarone (5 mg/kg) was administered after the fifth shock, if needed. Persistent shockable arrhythmias beyond that were subjected to subsequent shocks, along with 2 g magnesium and 100 mg lidocaine. All defibrillation attempts were made with the animal in antero-apical pad position.

Due to laminar blood flow induced by conventional ECC and the lack of invasive blood pressure and blood gas measurements, no information regarding blood pressure and blood gases were available in conventional ECPR during the first 60 min (prehospital situation). Consequently, no vasoactive drugs were applied and no active modification of blood gas parameters took place during the pre-hospital ECPR phase. In case of venous-cannula suction events, a bolus of 300–500 mL crystalloid fluid was administered.

In the second, in-hospital section (60–90 min), hypotension was treated with norepinephrine and crystalloid fluid administration to enable a mean arterial blood pressure (MAP) > 65 mmHg [24]. Arterial acidosis was counteracted by injecting sodium bicarbonate in order to achieve a pH greater than 7.2.

#### 2.5.2. Controlled Automated Reperfusion of the Whole Body (CARL)

In the CARL group, the CARL controller (Resuscitec GmbH, Freiburg, Germany) was used for reperfusion, and CARL therapy was applied [16,17,18]. In accordance with CARL therapy, more than 14 blood parameters and 4 specific reperfusion conditions were modified [16,25]. Before use, the system was primed with 1.0 L specially developed priming solution (CARL Priming Solution, Dr. Franz Köhler Chemie GmbH, Bensheim, Germany) [16]. Initial reperfusion was performed at a high, pulsatile blood flow of 80–100 mL/kg/min, and pulsatility was maintained for 45 min or until the heart regained its own ejection. As provided, a fibreoptic catheter (CARL Arterial Pressure Sensor, Resuscitec GmbH, Freiburg, Germany) was placed in the ascending aorta via the integrated connection site in the arterial line to monitor arterial pressure immediately after initiating reperfusion. A MAP of 80–100 mmHg was targeted by injecting norepinephrine and fluids (priming solution and crystalloid fluid). Immediate hypothermia up to 32 °C was induced in the first 60 min followed by gradual rewarming to normothermic body temperature. Mechanical ventilation was performed in a volume-controlled manner with one-third of the tidal volume required before, and the inspiratory oxygen concentration was set to 30% until the heart regained its own ejection. The sweep gas flow and oxygen supply via the membrane oxygenator was adjusted to achieve the target values required in CARL therapy (arterial P_O2_ 100–200 mmHg, arterial P_CO2_ 35–45 mmHg). By injecting sodium bicarbonate, a physiologic pH was targeted form the 45th min onwards.

Shockable cardiac arrhythmias during the reperfusion process were managed by injecting 40 mL potassium (7.45%) to evoke a transient asystole, and allow for a rhythm conversation into an organized hearth rhythm. This form of potassium-induced secondary cardioplegia was repeated once if the arrhythmia persisted before more conversion attempts were made with repeated electrical defibrillations at 200 joules. After the first electrical defibrillation, supportive 10 mg/kg amiodarone was administered via peripheral venous access, followed by another dose of 5 mg/kg after the third shock. If ventricular fibrillation persisted, additional 100 mg lidocaine and 2 g magnesium were applied. To further evaluate the effect of SC beyond the CARL therapy, it was also applied in n = 4 animals in the ECPR group showing refractory ventricular fibrillation (VF) at different time points.

### 2.6. Statistical Analyses

Statistics were analysed and data were visualised using the statistical software RStudio (version 1.4.11) and R package ggplot2 [26,27]. Data were tested for normal distribution by Shapiro–Wilk test. Means and standard deviation (SD) or medians and ranges were calculated as appropriate. Differences between groups were compared applying Mann–Whitney rank sum test or *t*-Test as appropriate. The number of defibrillations required was tested in a generalised linear model using Poisson Regression. Analysis of categorical data, such as cardiac arrhythmias, is limited to descriptive analysis due to small subgroup size. Statistical analyses regarding blood pressure and haemoglobin concentration were conducted in a mixed-effects model for each phase of the experiment using the R packages lme4 and lmerTest [28,29]. In this model, bodyweight, time of measurement and group allocation were considered as fixed effects. A group size of n = 10 in each group allowed the discrimination of differences with an effect size greater than 1.2, a power of 0.8 and a one-tailed significance value of 0.05.

## 3. Results

### 3.1. Haemoperfusion

In the CARL group, a mean blood flow of 5.26 ± 0.36 L/min was achieved compared to 3.12 ± 0.36 L/min in the ECPR group (*p* < 0.001). This required significantly more fluid administration in the CARL group over the entire reperfusion period than in the ECPR group (Table 1, CARL: 1556 ± 687 mL, ECPR: 430 ± 286 mL, *p* < 0.01).

**Figure 2:**
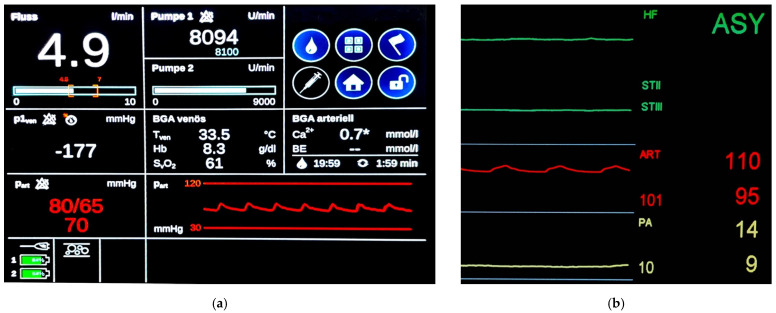
Pulsatile blood flow on ECC during CARL therapy: (**a**) Screenshot from real-time measurements on the CARL controller during asystole reperfusion. A pulsatile blood pressure was observed during extracorporeal circulation in the ascending aorta during asystole phase (red curve); (**b**) Screenshot of experimental monitoring during ECC. Despite asystole (green curve), a pulsatile blood flow (red curve) is detected in the common carotid. Pulmonary artery (PA) pressure is displayed in yellow.

The pulsatile blood flow generated by the CARL controller’s second diagonal pump could be tracked in the abdominal aorta as well as in the common carotid artery (Figure 2). As is standard with CARL therapy, we observed an amplitude of 15–20 mmHg in both vessels at a 50/min pulse rate.

We observed no complications in relation to pulsatility or the high flow approach such as aortic valve insufficiency or left ventricle distension.

### 3.2. Haemodynamics

**Figure 3:**
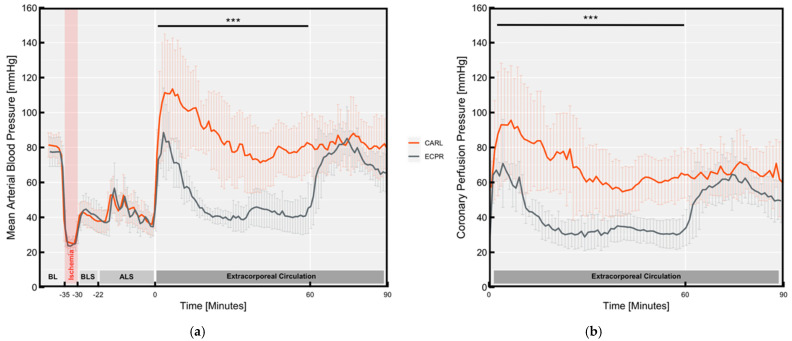
Longitudinal course of haemodynamic parameters: (**a**) Mean arterial blood pressure; (**b**) Coronary perfusion pressure. Data are expressed as mean ± SD. BL Baseline, BLS Basic life support, ALS Advanced life support. (*** *p* < 0.001).

Neither group differed significantly in MAP during baseline, ischaemia and mechanical resuscitation (see Figure 3a). A trend towards decreasing blood pressure was observed during the BLS and ALS phases, which was interrupted by repetitive peaks in the ALS phase. These peaks corresponded to the repeated application of epinephrine. However, this blood pressure-increasing effect exhibited a visibly decreasing course as the number of applications rose. The effect of the fifth application, two minutes before the start of ECC, coincided with the initial phase of reperfusion and contributed to a further blood pressure peak after extracorporeal reperfusion in both groups.

This initial peak revealed a rapid decline in accordance with the half-life of epinephrine (3–10 min) in the ECPR group. Due to the unavailability of prehospital arterial pressure measurements, we could not respond appropriately to this drop, resulting in a critical MAP less than 60 mmHg from the fifteenth minute on in all ten animals. At the beginning of the in-hospital phase after 60 min of ECC, we were able to treat low arterial pressure by injecting norepinephrine and fluids as in Table 1, resulting in a sufficient MAP of 72 ± 7 mmHg in the ECPR group.

The CARL group revealed a significantly higher MAP during the first 60 min of the experiment (pre-hospital phase). The combination of catecholamine and fluid application and the pulsatile, high flow approach resulted in a MAP of 87 ± 13 mmHg.

Concomitant with the CARL group’s higher MAP, they also achieved higher coronary perfusion pressures (CPPs) during the first hour of ECC than did the ECPR group (see Figure 3b, CARL: 69.3 ± 14.0 mmHg, ECPR: 36.8 ± 4.6 mmHg, *p* < 0.001). In the second reperfusion phase, CPPs in both groups were similar with the CARL group tending towards higher coronary pressures (CARL: 65.2 ± 12.5 mmHg, ECPR: 54.1 ± 8.7 mmHg, *p* = 0.10). Although both groups required similar numbers of vasopressor applications, the CARL group needed three times as much fluid.

**Figure 4:**
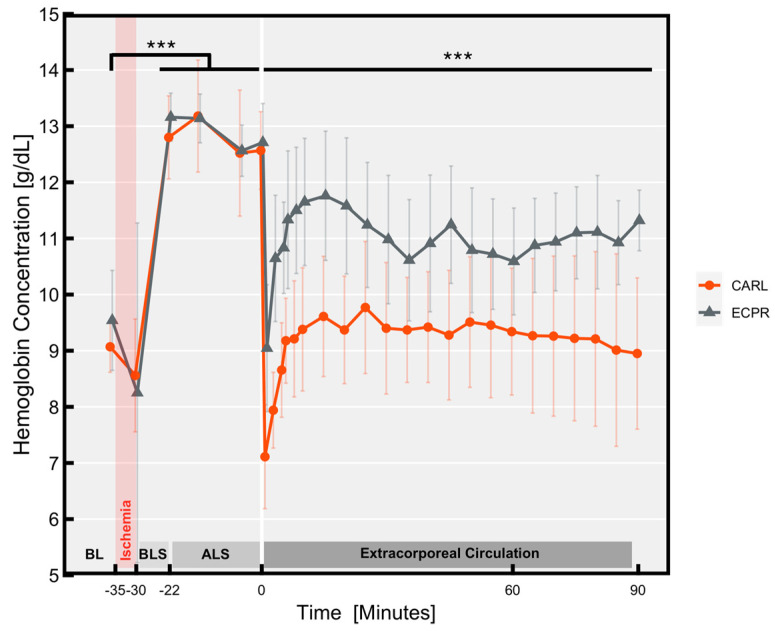
Haemoglobin concentration’s longitudinal course in arterial blood. Data are expressed as mean ± SD. BL Baseline, BLS Basic life support, ALS Advanced life support. (*** *p* < 0.001).

As shown in Figure 4, the haemoglobin concentration rose significantly during chest compressions compared to baseline (13.0 ± 0.7 g/dL vs. 9.3 ± 0.7 g/dL, *p* < 0.001), indicating a relevant haemoconcentration caused by volume shift during cardiac arrest. We detected no differences between the groups until the onset of reperfusion. After starting reperfusion, we noted a complete return to baseline values in the CARL group, but the ECPR group’s haemoglobin concentration did not entirely return to baseline values. Even after a prolonged period of reperfusion, they still differed significantly from the CARL group (*p* < 0.001). We found that animal body weight proved to be a significant confounder for haemoglobin concentrations during reperfusion (*p* = 0.044) with higher haemoglobin concentrations in heavier animals.

### 3.3. Arterial Blood Gases

Arterial blood gas analysis revealed no differences in baseline values and no differences at the end of the ALS phase between booth groups (see Table 2).

Arterial P_O2_ during ECC was higher in the ECPR group (*p* < 0.001), as well as arterial P_CO2_ (*p* < 0.01) in the pre-hospital phase. No differences were seen in lactate and glucose concentration between booth groups. Arterial pH showed a significant reduction in the ECPR group 30 (*p* < 0.05) and 60 (*p* < 0.001) minutes after the onset of ECC.

### 3.4. Effects on Rhythm Conversion and Cardiac Arrhythmias during Reperfusion

#### 3.4.1. Termination of Ventricular Fibrillation

Following ALS, all pigs exhibited persistent VF. With the onset of ECC in the CARL group, the reperfusion solution containing lidocaine and a high concentration of magnesium induced a transient asystole in all animals. Spontaneous rhythm conversion to sinus rhythm was observed in n = 4 animals (see Table 3). In two of those animals (2/4), the sinus rhythm proved to be stable until the end of the observation period. VF in the remaining n = 8 pigs was managed by potassium-induced secondary cardioplegia (SC) as described above. All animals receiving SC showed a transient asystole phase interrupted by either sinus rhythm or further cardiac arrhythmias. Sustained asystole after SC was not observed in any animal. The sinus rhythm achieved in one pig (1/8) after SC appeared to be stable until the end of the observation period. A mean increase in serum potassium concentration of 0.8 ± 0.1 mmol/L per application was observed. The remaining pigs (7/10) with shockable arrhythmias underwent further defibrillation attempts in accordance with the guidelines.

To further evaluate the SC effect beyond CARL therapy, we also evaluated it in n = 4 animals in the ECPR group suffering from refractory VF despite several defibrillations (range 9–13 attempts) at different time points. SC was applied in n = 2 animals during the pre-hospital phase with a MAP of 33 and 36 mmHg, respectively, during administration, another n = 2 animals received SC during the in-hospital phase with a MAP of 58 and 87 mmHg during administration. All animals (4/4) showed a transient asystole followed by persistent sinus rhythm until the end of the observation period. No further cardiac arrhythmias were observed in those animals.

#### 3.4.2. Number of Defibrillations

**Figure 5:**
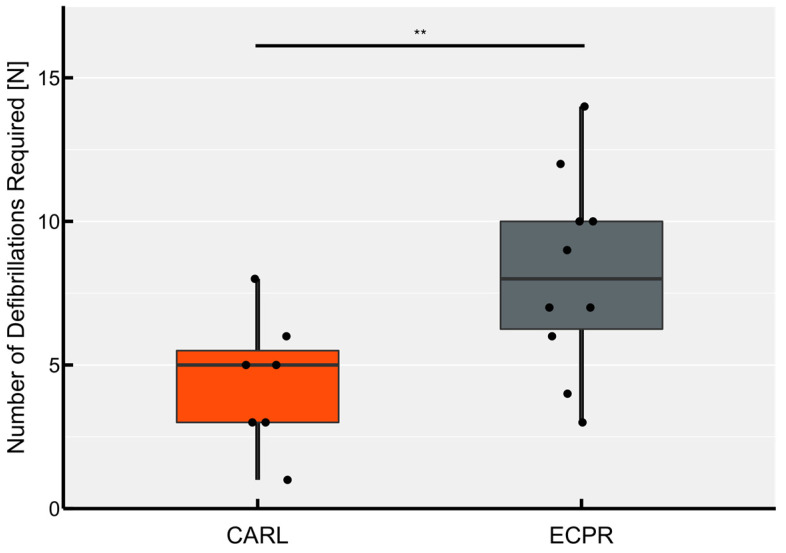
Number of shocks (200 J) delivered. Each point represents the number of electric defibrillations required per animal for sustained rhythm conversion to sinus rhythm (** *p* < 0.01).

In the ECPR group, a median of 8 (range 3–14) electrical defibrillation attempts were necessary to achieve sustained rhythm stability, compared to 5 (range 1–8) attempts in the CARL group (see Figure 5). This difference was statistically significant (*p* < 0.01) and remained significant even when considering the treatment attempts with secondary cardioplegia (median CARL 6 (range 1–10), ECPR 8.5 (range 3–15), *p* < 0.05).

#### 3.4.3. Cardiac Arrhythmias

Wide QRS complex bradycardias were observed in both groups after initial VF conversion (see Table 4). All bradycardias required therapeutic interventions, except for one animal in the ECPR group. Internal over-pacing at a rate of 100–120/min via the implanted pacemaker catheter was performed to maintain cardiac output.

Tachycardiac arrhythmias were documented in two animals, including a bigeminus rhythm in one pig and pulseless ventricular tachycardia (pVT) in two others. Defibrillation was performed for pVT, and the bigeminus rhythm appeared to be self-limiting.

All documented cardiac arrythmias in the ECPR group occurred before the application of secondary cardioplegia. We identified no significant differences in potassium levels between animals with or without cardiac arrhythmias (arrhythmias: 5.22 ± 0.57 mmol/L, no arrhythmias: 5.17 ± 0.93, *p* = 0.544).

## 4. Discussion

ECPR has been gaining increasing acceptance in the treatment of refractory cardiac arrest. Prolonged low-flow as that accompanying conventional CPR prior to ECPR is known to worsen the neurologic outcome after CA [30]. More and more pre-hospital ECPR programs have been launched to minimise the duration of low-flow. Over the last few decades, a concept specialized in pre-hospital ECPR named CARL was developed. By recording and modifying the most important reperfusion parameters, clinicians aim to achieve the greatest possible reduction in reperfusion damage using CARL [16,17,18,25].

The purpose of the present study was to investigate whether a pulsatile, high blood-flow approach is feasible when applying CARL therapy, and whether any effects on rhythm control appear in a reality-based porcine model of prolonged CPR. The rationale of this approach and effects on heart rhythm control compared to conventional ECPR are discussed below.

### 4.1. High Pressure and Pulsatile, High-Flow Reperfusion

It is well known that the cerebral no-reflow phenomenon is exacerbated in proportion to the duration of ischaemia, and that it can be reversed by raising reperfusion pressure [31,32]. Clinical studies have shown that high MAP and CPP are, together with high systolic blood pressure, associated with lower mortality and better neurological outcomes [33,34,35]. Moreover, dysfunctional cerebral autoregulation is often observed after CA, necessitating higher perfusion pressures to ensure adequate perfusion of all regions in the central nervous system [36]. Due to the unavailability of pre-hospital blood pressure monitoring on ECC, we observed relevant hypotension in the ECPR group. As shown in a systematic review by Bhate et al., the presence of hypotensive phases after ROSC is associated with increased mortality [35]. We detected no hypotensive phases in our CARL group, a finding that again highlights the importance of monitoring key reperfusion parameters including blood pressure as early as possible.

Higher perfusion pressures are also achievable especially on ECC by increasing blood flow. Together with pulsatile blood flow, beneficial effects on survival and neurological outcome have been described in conjunction with CARL therapy before [18]. In the present study we observed that even when using comparatively small cannulas, the pulsatility generated by the CARL controller can be maintained in the vascular system downstream in the smaller blood vessels. Despite the extreme haemoconcentration that occurs after prolonged cardiac arrest, we found this pulsatile, high-flow, high-pressure approach to be feasible.

Haemoconcentration occurs as a result of blood plasma redistributed from the intravascular to the extravascular space due to failure of the energy-dependent sodium–potassium pump combined with enhanced cell permeability and increased intracellular osmolarity [37]. Beyond that, the compression of upper abdominal organs during chest compression contributes to the rise in the haemoglobin concentration. However, haemoconcentration and accompanying increased blood viscosity have been proven to trigger impaired cerebral circulation after ischaemia [32]. As our experiments showed, a haemoconcentrated state raises the fluid demand, which was adequately fulfilled in the CARL group. More research is needed to determine whether additional haemodilution beyond baseline values reveals beneficial effects regarding cerebral blood flow and functional outcome in conjunction with CARL therapy.

### 4.2. Impact on Rhythm Conversion and Cardiac Arrhythmias

As mentioned above, CARL therapy takes a modified approach for rhythm conversion in shockable arrhythmias via established cardiac surgery techniques [16,19,20]. The combination of lidocaine–magnesium-induced and potassium-induced cardiac standstill led to sustained rhythm conversion in 30% of all animals not requiring any electrical defibrillation, encouraging evidence in light of the potential harmful side effects of defibrillations such as electrical and contractile dysfunction [38,39,40]. Even when cardioplegia failed, thus requiring defibrillation, we observed that the number of defibrillations required was significantly reduced using CARL. There is ample evidence of the positive effects of high MAP and CPP on the efficacy of defibrillation and survival [33,34]. It is thus likely that the effects we observed can be at least partially attributed to the CARL group’s higher CPP. In their study, Lee et al. also demonstrated a decrease in the number of countershocks required after potassium-induced cardiac standstill. Interestingly, their groups’ MAP and CPP were similar, suggesting a CPP-independent effect [41]. Furthermore, defibrillation and rhythm stability may be facilitated by a more rapid recovery of the acid–base balance as well as the more physiologic oxygen and carbon dioxide partial pressures during CARL therapy.

As our findings suggest, potassium-induced secondary cardioplegia is effective regardless of CARL therapy, as indicated by its efficacy in four ECPR-group animals. As shown in two animals, SC’s anti-arrhythmic effect remains intact even at lower perfusion pressures close to those at the end of the ALS phase, which raises the question of SC’s role and effectiveness in conventional CPR. Lee et al. demonstrated beneficial effects of potassium and potassium–lidocaine-induced cardiac standstill even in conventional CPR without employing ECC [41,42]. In their study, animals receiving potassium required fewer countershocks, smaller doses of adrenaline, and their CPR was of shorter duration [41]. However, it is well known that inducing cardiac standstill in the clinical routine, regardless of how it is implemented, should only be performed when stable circulatory support is available via extracorporeal circulation.

It should also be noted that repeated applications of secondary cardioplegia are known to raise the concentration of serum potassium. This may become relevant when considering hyperkalaemia as an accompanying consequence or even possible cause of CA [6]. In this situation, the additional administration of potassium could be associated with an increase in potentially harmful bradyarrhythmias and, thus, worsen CA outcome. We observed no increase in bradyarrhythmias in our study despite repeated applications of potassium-induced cardioplegia. On the contrary, applying CARL therapy, we detected a decrease in cardiac arrhythmias. Moreover, employing a point-of-care blood gas analyser or the CARL controller with its integrated arterial blood gas analyser enables patient-specific therapy due to the immediate availability of electrolyte measurements. This in turn enables diagnosis of pre-existing hyperkalaemia and immediate treatment after the onset of reperfusion. Potassium-induced secondary cardioplegia should be avoided in this case.

### 4.3. Limitations

Our study has several limitations. In our model, CA was induced electrically and not by any organic pathology. Moreover, to prevent premature ROSC, no amiodarone and no defibrillations were allowed during conventional CPR.

For logistical reasons, as we were unable to extend the observation period, the ECC phase, especially, turned out to be rather short. Longer observation periods are obviously needed to assess long-term cardiac effects. Furthermore, we only investigated myocardial factors in this study, whereas the limitation of neurological damage needs to be investigated in further studies.

Moreover, cardiac IRI occurs as a result of multiple mechanisms impacting rhythm conversion and provoking cardiac arrhythmias. Most of these mechanisms, such as inflammatory response or microcirculatory deficits, were not considered in this study; although, it is well known that they play a crucial role in the development of cardiac IRI.

## 5. Conclusions

Our results indicate that despite the dramatically excessive haemoconcentration that occurs during cardiac arrest, CARL therapy makes high-pressure, pulsatile, high-flow reperfusion feasible. Life-threatening hypotension can be prevented during the pre-hospital phase of extracorporeal resuscitation by applying CARL. Moreover, controlling the heart rhythm via CARL reperfusion proved to be effective by minimizing defibrillation attempts and cardiac arrhythmias. Even spontaneous rhythm conversions were observed during CARL therapy. This suggests CARL therapy’s potential benefit in alleviating refractory OHCA, thus improving the outcomes of patients undergoing ECPR.

## Figures and Tables

**Table 1:** Norepinephrine and fluid use in reperfusion phase.

		Mean ± SD	
	n	Norepinephrine (µg/kg/min)	Fluid (mL)
CARL	8 *	1.15 ± 0.70	1556 ± 687
ECPR	10	1.82 ± 1.33	430 ± 286
*p*-Value		0.22	0.002

* No information on drug application available for n = 2 pigs (loss of data).

**Table 2:** Arterial blood gas analysis.

	ECPR							CARL					
	BL	ALS	3’	30’	60’	90’		BL	ALS	3’	30’	60’	90’
pH	7.45 ± 0.02	7.09 ± 0.16	7.01 ± 0.09	7.08 * ± 0.06	7.11 *** ± 0.08	7.29 ± 0.05		7.45 ± 0.02	7.07 ± 0.10	7.01 ± 0.05	7.17 * ± 0.08	7.29 *** ± 0.08	7.33 ± 0.06
Base excess, mmol/L	3.6 ± 1.2	−14.4 ± 2.9	−15.1 * ± 3.5	−13.5 ± 1.9	−13.0 * ± 2.4	−5.8 ± 1.2		3.8 ± 1.3	−15.8 ± 3.3	−18.4 * ± 1.6	−14.9 ± 4.1	−9.1 * ± 4.2	−6.2 ± 2.9
Arterial P_O2_, mmHg	93 ± 17	110 ± 140	207 ± 82	233 *** ± 71	255 *** ± 94	424 *** ± 104		91 ± 7	71 ± 30	224 ± 64	118 *** ± 31	103 *** ± 29	115 *** ± 41
Arterial P_CO2_, mmHg	40 ± 1	56 ± 24	64 ** ± 9	58 *** ± 10	55 ** ± 10	43 ± 7		41 ± 2	54 ± 25	53 ** ± 6	39 *** ± 7	37 ** ± 8	38 ± 5
Glucose, mg/dL	98 ± 20	276 ± 168	305 ± 164	238 ± 163	201 ± 140	171 ± 141		102 ± 9	381 ± 79	296 ± 76	234 ± 68	226 ± 61	219 ± 52
Lactate, mmol/L	1.6 ± 0.4	10.7 ± 2.5	12.0 ± 2.1	12.0 ± 1.2	12.1 ± 1.6	14.7 ± 1.7		1.5 ± 0.5	10.9 ± 1.6	10.2 ± 1.2	11.4 ± 1.6	13.2 ± 1.7	14.0 ± 1.7

Values are expressed as mean ± SE. BL Baseline, ALS End of advanced life support. (* *p* < 0.05, ** *p* < 0.01, *** *p* < 0.001).

**Table 3:** Rhythm conversation procedure.

	Spontaneous Rhythm Conversion		Secondary Cardioplegia		Electric Defibrillation	
Group	With Reperfusion (n)	Sustained (n)	Required (n)	Attempts Median (min, max)	Required (n)	No. of Shocks Median (min, max)
CARL	4/10	2/4	8/10	2 (1,2)	7/10	5 (1,8)
ECPR	0/10	0/0	4/10	1 (1,1)	10/10	8 (3,14)

**Table 4:** Cardiac arrhythmias during reperfusion.

	Bradyarrhythmia		Tachyarrhythmia	
Group	n	Therapy-Needed	n	Therapy-Needed
CARL	1/10	1/1	1/10	1/1
ECPR	4 */10	3/4	2 */10	1/2

* One animal exhibited bradyarrhythmia and tachyarrhythmia.
